# Multimodal deep learning integrating ultrasonographic and clinical data for enhanced rotator cuff tear identification

**DOI:** 10.3389/fphys.2026.1884047

**Published:** 2026-09-11

**Authors:** Ye Jiang, Huining Xu, Yuxuan Hu, Mengyao Xu, Jinping Wang, Tianyou Xin

**Affiliations:** Department of Ultrasound, Wuxi Ninth People’s Hospital Affiliated to Soochow University, Wuxi, Jiangsu, China

**Keywords:** deep learning, diagnostic accuracy, explainable artificial intelligence, multimodal fusion, rotator cuff tear, ultrasonography

## Abstract

**Background:**

Ultrasonography (US) represents a practical and accessible imaging modality for rotator cuff tear (RCT) evaluation, yet diagnostic accuracy remains constrained by operator experience and the sonographic ambiguity of partial-thickness tears. Deep learning (DL) models applied to US imaging and machine learning models derived from clinical variables have each demonstrated independent promise, yet their integration within a unified diagnostic framework has not been systematically explored.

**Objective:**

This study aimed to develop and validate a multimodal DL model integrating US imaging with structured clinical examination data for RCT detection.

**Methods:**

A retrospective cohort of 947 patients presenting with shoulder pain who underwent both shoulder US and MRI was partitioned into training and testing sets at a 7:3 ratio. Patients with MRI-confirmed supraspinatus RCT were designated RCT-positive, while those with supraspinatus tendinopathy or subacromial bursitis were designated RCT-negative. Four model configurations were developed: a clinical-only model, a single-view US model, a dual-view US model, and a full multimodal model (MM) combining dual-view US with clinical variables through feature-level fusion. All models were trained using five-fold cross-validation and evaluated on the testing set for discriminative performance, calibration, and clinical utility. Model interpretability was assessed using Gradient-weighted Class Activation Mapping (Grad-CAM) for the image encoder and SHapley Additive exPlanations (SHAP) for the clinical encoder.

**Results:**

Among 947 patients, 370 (39.1%) were RCT-positive and 577 (60.9%) were RCT-negative. MM achieved the highest area under the receiver operating characteristic curve (AUROC) of 0.935, with sensitivity of 0.946, specificity of 0.786, and F1 score of 0.830, significantly outperforming all unimodal configurations. Decision curve analysis confirmed superior net benefit of MM across clinically relevant threshold probabilities. Grad-CAM heatmaps demonstrated anatomically coherent attention within the supraspinatus tendon, while SHAP analysis identified the drop arm test, external rotation lag sign, and empty can test as the dominant clinical predictors.

**Conclusion:**

The proposed multimodal DL framework offers a validated and interpretable tool for MRI-independent RCT diagnosis, with potential applicability in resource-constrained clinical settings.

## Introduction

Rotator cuff tears (RCTs) are among the most prevalent musculoskeletal disorders in adults, with population-based studies reporting a prevalence of approximately 20% of the general population (Minagawa et al., 2013), rising to over 25% in individuals aged 70 years and above (Yamamoto et al., 2010). More recent evidence has further shown that RCTs and imaging-detected rotator cuff abnormalities are common across adult populations, including asymptomatic individuals (Hinsley et al., 2022; Sanders et al., 2025). In China, where the proportion of adults aged 60 years or older is projected to exceed 30% by 2035, the clinical burden of RCTs is expected to increase substantially (Gong et al., 2012). RCTs account for 5 to 40% of all shoulder disorders and represent a leading cause of shoulder-related disability, pain, and diminished quality of life (Zhao et al., 2023). Accurate diagnosis is therefore a prerequisite for effective management, as RCTs carry fundamentally different treatment implications compared with other common supraspinatus region pathologies. In clinical practice, patients presenting with shoulder pain or dysfunction represent a diagnostically heterogeneous population in whom RCTs must be carefully distinguished from supraspinatus tendinopathy (SST) and subacromial bursitis (SAB). Although these conditions share overlapping clinical presentations and may generate ambiguous sonographic findings, SST and SAB are generally managed conservatively, whereas RCTs may ultimately require surgical intervention (Plancher et al., 2021; Nalbant and Inci, 2023; Al Khayyat et al., 2024; Garcia et al., 2024). Failure to accurately differentiate RCTs from these entities therefore risks both undertreatment of surgically correctable pathology and unnecessary procedural exposure, underscoring the clinical imperative for precise and reliable diagnostic strategies.

Magnetic resonance imaging (MRI) is currently regarded as the preferred imaging modality for RCT diagnosis, offering high-resolution multiplanar visualization of tendon integrity and associated osseous structures (Zoga et al., 2021). Its clinical utility, however, is constrained by high cost, lengthy acquisition times, limited availability in primary care settings, and patient-related barriers including ferromagnetic implants and claustrophobia (Zoga et al., 2021; Gowda et al., 2024). These barriers are particularly pronounced in China, where MRI resources remain unevenly distributed across regions, frequently delaying diagnosis and restricting access for a substantial proportion of patients. Ultrasonography (US) has emerged as a cost-effective and widely accessible alternative. Two large systematic reviews and meta-analyses demonstrated that US achieves a sensitivity of 0.88 and specificity of 0.93 for full-thickness rotator cuff tears (FTRCTs), with no statistically significant difference in diagnostic accuracy compared to MRI (Smith et al., 2011; Farooqi et al., 2021). Among all RCTs, however, partial-thickness rotator cuff tears (PTRCTs) are generally more prevalent than FTRCTs, yet they represent the subtype most prone to diagnostic error on US, with median sensitivity falling to only 0.65 (Ardeljan et al., 2020; Radhakrishnan et al., 2024). This limited sensitivity is attributed to the variable echogenicity of peritendinous tissue, which can obscure clear differentiation between torn and intact tendon fibers at the lesion margin (Ok et al., 2013; Farooqi et al., 2021), and the irreducible influence of operator experience (Teefey et al., 2000; Iannotti et al., 2005). The limitation carries direct clinical consequence, given that a substantial proportion of untreated PTRCTs defects progress to FTRCTs within three years (Thangarajah and Lo, 2022). These findings collectively underscore that single-modality US, as currently practiced, cannot fully replace MRI, particularly in diagnostically ambiguous cases involving partial-thickness pathology.

Recent advances in artificial intelligence (AI) present a promising avenue for addressing these diagnostic limitations. Deep learning (DL) models applied to static US images have demonstrated considerable accuracy in detecting rotator cuff pathology, with certain attention-based architectures additionally shown to enhance interobserver reliability among clinicians (Wu et al., 2025). Separately, machine learning models derived exclusively from clinical examination variables, encompassing physical test findings, range of motion parameters, and patient demographic characteristics, have exhibited strong discriminative performance in RCT prediction (Li et al., 2023). These parallel lines of evidence suggest that both imaging features and clinical variables independently carry substantial predictive signal. However, studies specifically aimed at improving US-based identification of RCTs through AI remain scarce.

The present study addresses this gap by developing and validating a multimodal DL model that integrates US features with structured clinical variables for RCT detection, with MRI serving as reference standards. The integration of objective morphological evidence derived from US with clinically obtained predictive information holds considerable potential to improve diagnostic performance beyond the capacity of either data source in isolation, thereby offering a more accessible and clinically viable diagnostic strategy in resource-constrained settings where advanced imaging modalities remain unavailable or impractical.

## Materials and methods

### Ethics statement

This retrospective study was approved by the Institutional Review Board of Wuxi Ninth People’s Hospital Affiliated to Soochow University (KS2026-058-01) and conducted in accordance with the principles outlined in the Declaration of Helsinki. Given the retrospective nature of the study and the use of fully deidentified data, the requirement for informed consent was waived.

### Patient selection

We identified patients who underwent shoulder US at Wuxi Ninth People’s Hospital Affiliated to Soochow University between January 2022 and April 2026, yielding an initial pool of 1,500 candidates. Patients were eligible if they met all of the following criteria: age ≥18 years; presentation with shoulder pain or dysfunction lasting at least two weeks; completion of a standardized shoulder US examination; an MRI of the affected shoulder performed within 60 days of the index US, with a confirmed diagnosis of supraspinatus RCT, SST, or SAB as determined by board-certified radiologists; availability of complete clinical data including symptom duration, physical examination findings, and relevant demographic characteristics. Of the 1,500 candidates screened, 1192 met these inclusion criteria. Patients were excluded if they had a history of ipsilateral shoulder surgery (n=112), inflammatory arthropathy such as rheumatoid arthritis or crystalline arthropathy (n=58), acute fracture or dislocation at presentation (n=24), systemic neuromuscular conditions affecting shoulder function (n=16), or inadequate US image quality (n=35), defined as incomplete visualization of the supraspinatus tendon or severe acoustic shadowing or anisotropy that obscured the tendon margin. After applying these criteria, a final cohort of 947 patients was retained for analysis.

### Data collection

All shoulder US examinations were performed using a GE Logiq E20 system equipped with a high-frequency linear transducer (10–15 MHz) by one of three musculoskeletal sonographers each with a minimum of five years of dedicated shoulder imaging experience, following a standardized dynamic scanning protocol. Each examination systematically evaluated the supraspinatus, infraspinatus, subscapularis, and long head of the biceps tendons in both longitudinal and transverse planes, with dynamic maneuvers applied where indicated. Sonographic findings recorded included tendon continuity, the presence and location of hypoechoic or anechoic defects, bursal thickening or effusion, cortical irregularity of the humeral head, and calcific deposits.

Structured clinical variables were extracted from electronic medical records at the time of the index US examination. Demographic and anthropometric data included age, sex, body mass index (BMI), affected side, and symptom duration. Symptom duration was recorded in weeks as a continuous variable and was defined as the interval from patient-reported symptom onset to the index US examination. Trauma-related onset was coded as a binary variable and was defined as shoulder pain or dysfunction beginning after an identifiable acute event, such as a fall, direct impact, traction injury, or sudden lifting injury. Pain severity was assessed using the visual analogue scale (VAS), ranging from 0 to 10, with 0 indicating no pain and 10 indicating the worst imaginable pain. Active range of motion measurements in forward flexion, abduction, and internal and external rotation were retrieved in degrees. Physical examination findings comprised results from the Neer impingement sign, Hawkins–Kennedy test, empty can test, drop arm test, and external rotation lag sign, each recorded as positive or negative.

### Data preprocessing

Each patient was assigned a binary outcome label based on MRI findings. Patients with MRI-confirmed supraspinatus RCT were designated RCT-positive, and those with SST or SAB were designated RCT-negative. The cohort was then partitioned into training and testing sets at a ratio of 7:3 using stratified random sampling based on the outcome label.

Raw ultrasound images were converted to PNG format and cropped to remove peripheral user-interface elements, retaining only the active B-mode scan field. Cropped images were three-channel arrays with consistent pixel values across channels and were used as input to the EfficientNet-B4 backbone. Images were zero-padded to preserve the original aspect ratio and resized to 224 × 224 pixels using bilinear interpolation. This input size was selected to harmonize images with heterogeneous original dimensions and to support stable end-to-end training while limiting computational burden and overfitting risk in the available cohort. Pixel values were scaled to the range of 0–1 and normalized using the ImageNet channel-wise mean and standard deviation. Training images were augmented using random horizontal flipping, small-angle rotation, slight translation and scaling, and brightness and contrast jittering, whereas validation and testing images underwent deterministic preprocessing only. Image preprocessing was performed in Python 3.14.3 using OpenCV 4.13.0.92 for cropping, padding, and resizing and torchvision 0.27.1 for normalization and data augmentation.

Missing values in clinical variables were imputed using the median for continuous variables and the mode for categorical variables. Continuous clinical variables were standardized to zero mean and unit variance. Binary categorical variables, including sex, affected side, trauma-related onset, and physical examination findings, were encoded as 0 or 1; no non-binary categorical variables were included. Missing-value imputation and feature standardization parameters were estimated exclusively from the training data. During five-fold cross-validation, these parameters were estimated from each fold-specific training subset and applied to the corresponding validation subset. For final testing, parameters estimated from the full training set were applied unchanged to the held-out testing set.

### DL model development

**Figure 1:**
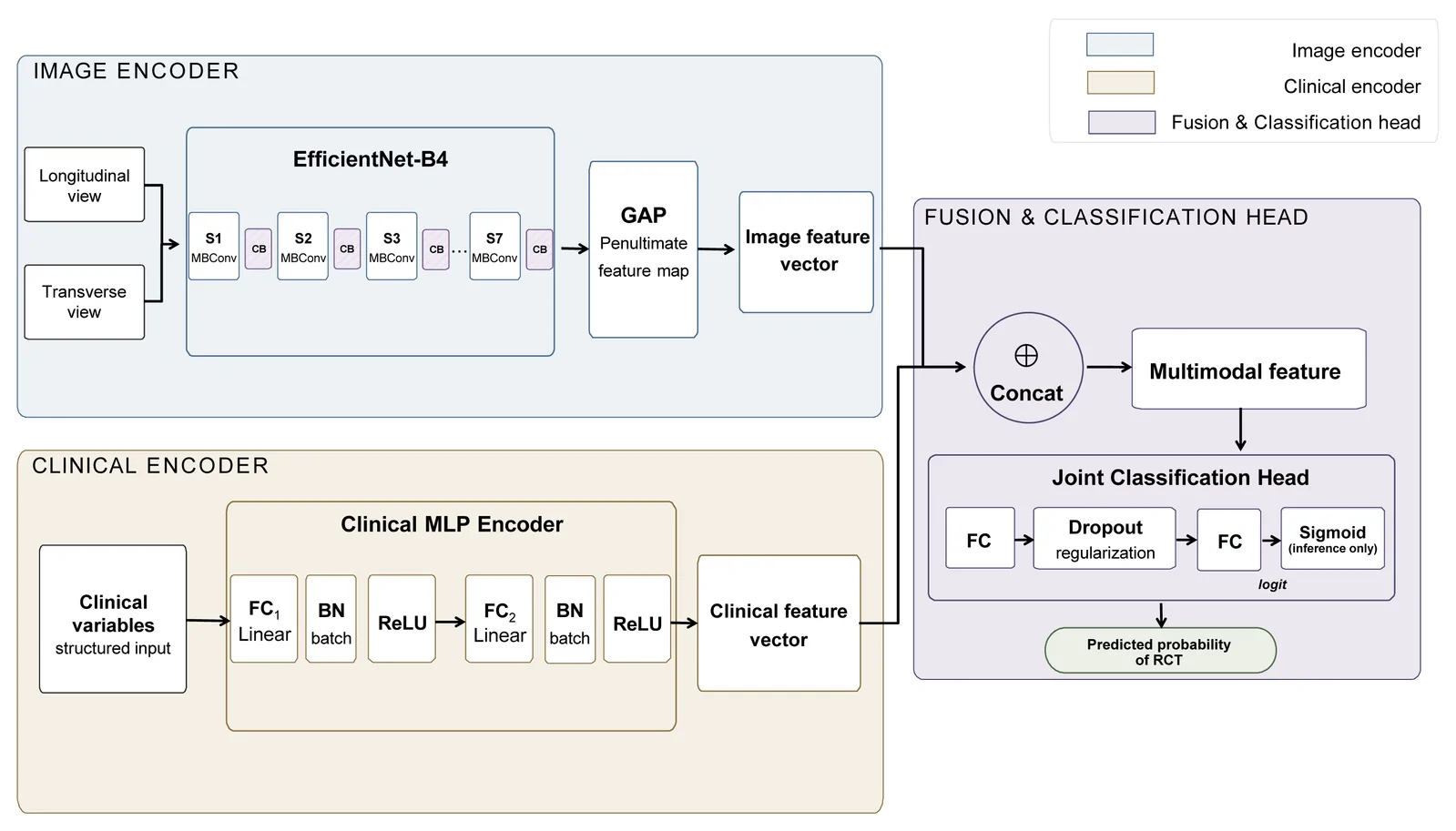
Architecture of the multimodal DL framework for RCT detection. The image encoder processes US images through an EfficientNet-B4 backbone comprising seven stages (S1–S7) of mobile inverted bottleneck convolution (MBConv) blocks, each followed by a CBAM, with global average pooling (GAP) applied to the penultimate feature map to generate an image feature vector. The clinical encoder processes structured clinical variables through a multilayer perceptron (MLP) comprising two fully connected (FC) linear layers, each followed by batch normalization (BN) and rectified linear unit (ReLU) activation, yielding a clinical feature vector. The two vectors are concatenated (⊕) and forwarded through a joint classification head with dropout regularization, producing a raw logit during training, with a sigmoid activation applied only at inference to obtain the final predicted probability of RCT.

A multimodal DL framework was developed to jointly process US images and structured clinical variables through a feature-level fusion strategy (**Figure 1**). The image processing branch employed an EfficientNet-B4 backbone pretrained on ImageNet (Russakovsky et al., 2015; Tan and Le, 2019), comprising seven sequential stages (S1–S7) of mobile inverted bottleneck convolution (MBConv) blocks, each followed by a Convolutional Block Attention Module (CBAM) (Woo et al., 2018) that sequentially applied channel and spatial attention mechanisms. All 947 patients in the final cohort had both longitudinal and transverse US images available. Each view was independently processed through the same backbone with shared weights, and the resulting feature vectors were concatenated to form a unified 3584-dimensional image representation prior to downstream fusion. Global average pooling was applied to the penultimate feature map to extract a compact image feature vector, yielding a 1792-dimensional representation per view.

In parallel, all 16 structured clinical variables were encoded through the clinical variable branch, which comprised a multilayer perceptron with two fully connected layers (16 to 64 and 64 to 32 units), each followed by batch normalization and ReLU activation, yielding a 32-dimensional clinical feature vector. The two feature vectors were concatenated to form a 3616-dimensional unified multimodal representation and forwarded through a joint classification head comprising two fully connected layers (3616 to 128 and 128 to 1), with the first layer followed by ReLU activation and dropout regularization (rate = 0.5). During training, the second layer produced a single raw logit, with a sigmoid function applied at inference to obtain the predicted probability of RCT.

The model was developed and trained end-to-end in PyTorch (version 2.12.1), using the Adam optimizer with a binary cross-entropy loss computed on raw logits (Li et al., 2024), a learning rate of 1×10^−4^, and a batch size of 32, following commonly adopted defaults for Adam-optimized CNN training. To address class imbalance, class weights were computed as the ratio of negative to positive samples within each training fold and applied to the positive class via the pos_weight parameter of PyTorch’s BCEWithLogitsLoss function. Five-fold cross-validation was performed within the training set to verify training stability and determine an appropriate training duration. Within each fold, training was capped at a maximum of 100 epochs, with early stopping triggered if validation loss failed to improve by at least 1×10^−4^ for 10 consecutive epochs, and the model weights corresponding to the lowest validation loss were retained. The mean epoch at which early stopping occurred across the five folds was adopted as the training duration for final model development, and a final model was retrained from scratch on the complete training set using this configuration before being evaluated on the held-out testing set as the independent assessment.

### Model interpretability

Model interpretability was evaluated across both input modalities for the best-performing model configuration. For the image encoder, Gradient-weighted Class Activation Mapping (Grad-CAM) was applied separately to the longitudinal and transverse views, using gradients backpropagated from the model’s final output to the final convolutional layer of the shared EfficientNet-B4 backbone, generating view-specific spatial activation heatmaps superimposed onto the original US images to visualize regions most influential to the model’s prediction. For the clinical encoder, feature importance was assessed using SHapley Additive exPlanations (SHAP), implemented via the DeepExplainer method, with mean absolute SHAP values computed across the testing set to quantify the relative contribution of each clinical variable to model output.

### Comparative model evaluation

To assess the independent and incremental contributions of each input modality, four model configurations were developed and evaluated under identical training conditions and hyperparameter settings. The clinical-only model (Clin) processed structured clinical variables through the MLP encoder without any image input. The longitudinal US model (US-L) processed longitudinal images through the EfficientNet-B4 backbone with CBAM, using only the longitudinal-view feature vector from this same dual-view cohort to isolate the incremental contribution of the transverse view. The dual-view US model (US-LT) integrated both longitudinal and transverse images through the encoding strategy described above. The full multimodal model (MM) combined dual-view US with structured clinical variables. All four configurations were evaluated on the same testing set. Discriminative performance was assessed using the AUROC, sensitivity, specificity, accuracy, positive predictive value (PPV), negative predictive value (NPV), and F1 score, with the optimal classification threshold determined by maximizing the Youden index on pooled out-of-fold predictions from the training-set cross-validation and applied unchanged to the testing set. For the MM model, sensitivity was additionally examined separately for PTRCTs and FTRCTs to determine whether detection performance differed by tear severity. Calibration was evaluated using the Brier score and calibration curves. Clinical utility was assessed through decision curve analysis (DCA), quantifying the net benefit of each configuration for guiding clinical decision-making in patients with suspected RCT, across a range of clinically plausible threshold probabilities.

### Statistical analysis

All statistical analyses were performed using Python (version 3.14.3). Univariate comparisons of clinical variables between RCT-positive and RCT-negative patients were performed using independent-samples t-tests or Mann–Whitney U tests for continuous variables and chi-square or Fisher’s exact tests for categorical variables, as appropriate. Normality of continuous variables was assessed using the Shapiro-Wilk test, with t-tests applied to normally distributed variables and Mann-Whitney U tests applied otherwise; Fisher’s exact test was used in place of the chi-square test when any expected cell count was less than 5. These univariate analyses were conducted only to describe baseline differences between groups and were not used for clinical feature selection.

The 95% confidence interval (95% CI) for AUROC was estimated using 1,000-iteration bootstrap resampling. For the subgroup sensitivity analysis, 95% CIs were estimated using the Wilson score method. Pairwise differences in AUROC between model configurations were assessed using the DeLong test. A two-sided P value of less than 0.05 was considered statistically significant for this comparison.

## Results

### Patient characteristics

**Table 1** presents the baseline characteristics of all 947 patients stratified by RCT status. Missingness across clinical variables was low and fairly uniform, supporting the appropriateness of median and mode imputation without material impact on model training. Of these, 370 (39.1%) were RCT-positive and 577 (60.9%) were RCT-negative, the latter comprising patients with MRI-confirmed SST or SAB. Among RCT-positive patients, 246 (66.5%) had PTRCT and 124 (33.5%) had FTRCT. RCT-positive patients were significantly older, reported longer symptom duration, and more frequently described a trauma-related onset compared with RCT-negative patients. Forward flexion and abduction were both significantly more restricted in the RCT-positive group. Among the tendon-specific clinical tests, the empty can test, drop arm test, and external rotation lag sign each demonstrated substantially higher positivity rates in RCT-positive patients (all P<0.001).

**Table 1:** Baseline characteristics of the study cohort stratified by rotator cuff tear status.

Variable	Missing values, n (%)	Overall (n = 947)	RCT-positive (n = 370)	RCT-negative (n = 577)	*P* value
Age, years	0 (0.0)	55.3 ± 10.4	59.1 ± 9.6	52.8 ± 10.4	<0.001
Male sex, n (%)	0 (0.0)	444 (46.9)	181 (48.9)	263 (45.6)	0.321
BMI, kg/m^2^	20 (2.1)	24.8 ± 3.3	24.9 ± 3.3	24.7 ± 3.2	0.357
Right side affected, n (%)	2 (0.2)	617 (65.2)	248 (67.0)	369 (64.0)	0.345
Symptom duration, weeks	26 (2.7)	16 (8–34)	22 (10–48)	12 (6–26)	<0.001^a^
Trauma-related onset, n (%)	16 (1.7)	230 (24.3)	122 (33.0)	108 (18.7)	<0.001
VAS pain score	31 (3.3)	5.5 ± 1.9	5.6 ± 1.9	5.4 ± 1.9	0.114
Active range of motion					
Forward flexion, °	36 (3.8)	148.0 ± 21.8	141.2 ± 23.8	152.4 ± 19.6	<0.001
Abduction, °	35 (3.7)	140.0 ± 24.2	126.3 ± 26.8	148.7 ± 20.4	<0.001
Internal rotation, °	38 (4.0)	51.2 ± 12.2	50.8 ± 12.8	51.4 ± 11.6	0.392
External rotation, °	37 (3.9)	54.8 ± 13.7	54.1 ± 14.2	55.2 ± 13.3	0.234
Physical examination findings					
Neer impingement sign positive, n (%)	24 (2.5)	627 (66.2)	252 (68.1)	375 (65.0)	0.325
Hawkins–Kennedy test positive, n (%)	23 (2.4)	665 (70.2)	267 (72.2)	398 (69.0)	0.294
Empty can test positive, n (%)	22 (2.3)	463 (48.9)	267 (72.2)	196 (34.0)	<0.001
Drop arm test positive, n (%)	20 (2.1)	92 (9.7)	72 (19.5)	20 (3.5)	<0.001
External rotation lag sign positive, n (%)	21 (2.2)	75 (7.9)	61 (16.5)	14 (2.4)	<0.001

^a^ compared using the Mann–Whitney U test.

The cohort was subsequently partitioned into training (n=663) and testing (n=284) sets via stratified random sampling, maintaining identical RCT-positive proportions across both subsets. **Supplementary Table 1** confirms well-balanced distributions across all baseline characteristics between the two sets (all P>0.05). Among the 284 patients in the testing set, 111 (39.1%) were RCT-positive, comprising 74 (66.7%) with PTRCT and 37 (33.3%) with FTRCT, and 173 (60.9%) were RCT-negative, comprising 112 (64.7%) with SST and 61 (35.3%) with SAB.

### Model development

The clinical branch encoded all 16 structured clinical variables. Four model configurations were trained under identical conditions and hyperparameter settings within the training set using five-fold cross-validation, with all configurations achieving stable convergence without evidence of overfitting across folds. Progressive integration of additional input modalities yielded monotonic improvements in validation performance, with mean five-fold cross-validation AUROCs (± standard deviation) of 0.784 ± 0.016, 0.851 ± 0.014, 0.866 ± 0.012, and 0.941 ± 0.011 for the Clin, US-L, US-LT, and MM configurations, respectively, indicating consistent performance with limited variability across folds. The incremental gain from US-L to US-LT was modest, whereas the addition of structured clinical variables to form the MM model produced a more substantial improvement over the dual-view US configuration alone.

### Model performance

**Figure 2:**
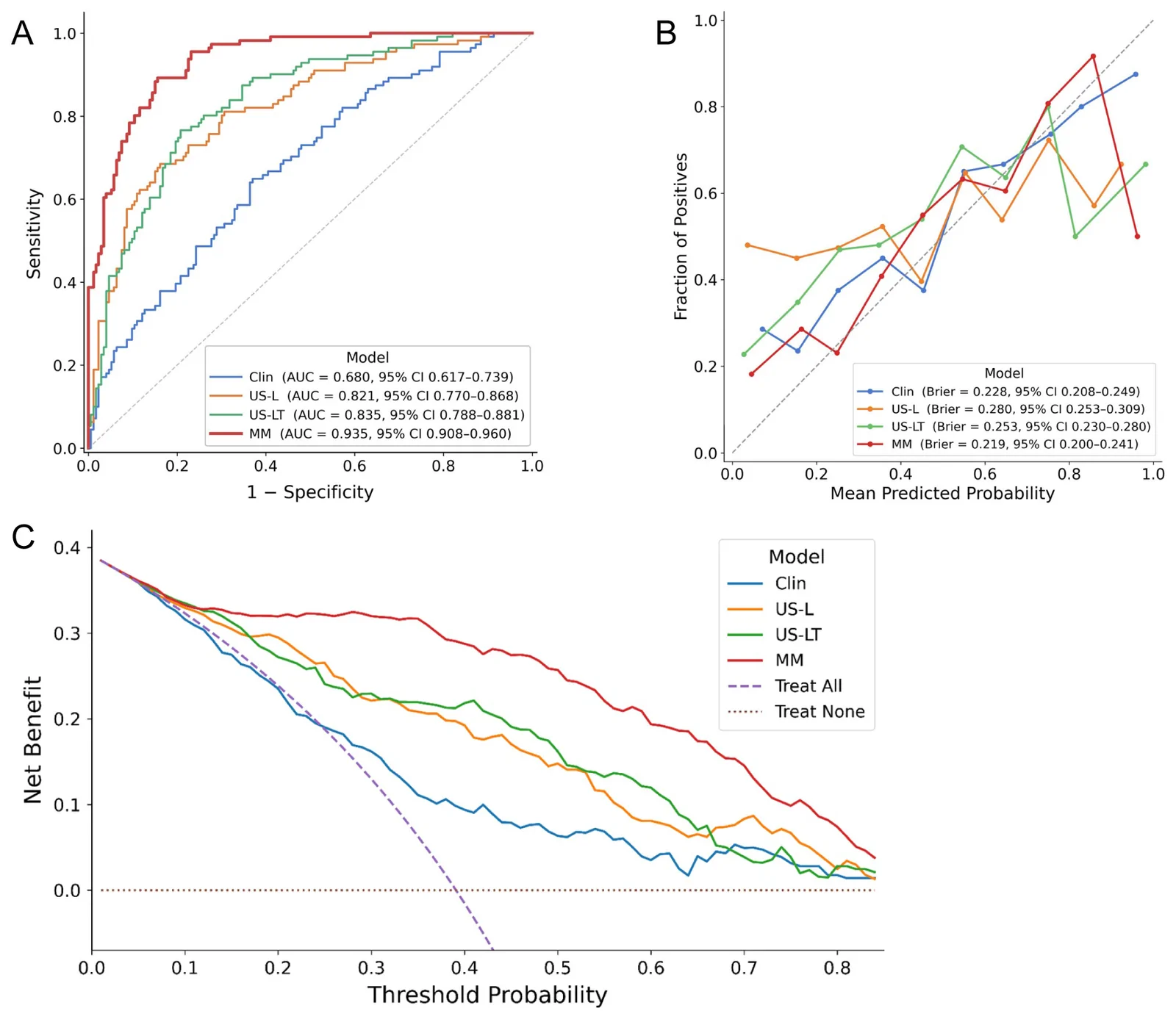
Comparative performance of the four model configurations on the testing set. **(A)** ROC curves with AUC and 95% CIs. **(B)** Calibration curves with Brier scores and 95% CIs. **(C)** DCA illustrating net benefit across threshold probabilities relative to treat-all and treat-none reference strategies.

ROC curve analysis revealed a consistent rank ordering across configurations, with MM occupying the most favorable position toward the upper-left corner, followed by US-LT, US-L, and Clin (**Figure 2A**). The separation between imaging-based models and Clin was pronounced, while the gap between US-L and US-LT was visually negligible. The AUROCs for Clin, US-L, US-LT, and MM were 0.680, 0.821, 0.835, and 0.935, respectively; pairwise DeLong tests for AUROC comparison confirmed that MM significantly outperformed US-LT, US-L, and Clin (all P < 0.001). The AUROC difference between US-LT and US-L was not statistically significant (P = 0.618), indicating that the addition of the transverse view alone conferred minimal discriminative gain. Both US-L and US-LT significantly outperformed Clin (both P < 0.001). At each configuration’s Youden-optimal classification threshold, MM achieved the highest sensitivity, specificity, accuracy, PPV, NPV, and F1 score among all configurations (**Table 2**). When the MM model was stratified by tear severity, every FTRCT was correctly classified, whereas all false-negative cases were confined to PTRCTs; subgroup sensitivity was accordingly lower for PTRCTs than for FTRCTs (**Table 3**).

**Table 2:** Diagnostic performance of the four model configurations on the testing set.

Model	Youden-optimal threshold	TP	FP	FN	TN	Sensitivity	Specificity	Accuracy	PPV	NPV	F1 score
Clin	0.460	72	64	39	109	0.649	0.630	0.637	0.529	0.736	0.583
US-L	0.350	90	60	21	113	0.811	0.653	0.715	0.600	0.843	0.690
US-LT	0.410	97	59	14	114	0.874	0.659	0.743	0.622	0.891	0.727
MM	0.430	105	37	6	136	0.946	0.786	0.849	0.739	0.958	0.830

**Table 3:** Sensitivity of the MM model on the testing set, stratified by rotator cuff tear thickness.

Subgroup	n	TP	FN	Sensitivity (95% CI)
PTRCT	74	68	6	91.9 (83.4–96.2)
FTRCT	37	37	0	100 (90.6–100)
Overall	111	105	6	94.6 (88.7–97.5)

Calibration curves demonstrated irregular deviation from the ideal diagonal across all models, particularly at higher predicted probability ranges (**Figure 2B**). MM achieved the lowest Brier score (0.219) relative to Clin (0.228), US-L (0.280), and US-LT (0.253), indicating comparatively better, though still imperfect, calibration among the four configurations. DCA showed that MM consistently yielded the highest net benefit across the widest range of clinically plausible threshold probabilities, sustaining superiority over the treat-all strategy at thresholds where all other models had converged toward or fallen below the treat-none reference line (**Figure 2C**). At the pre-specified operating threshold for MM (0.430), net benefit exceeded both the treat-all and treat-none strategies, supporting its potential to guide clinical decision-making.

### Model interpretability

**Figure 3:**
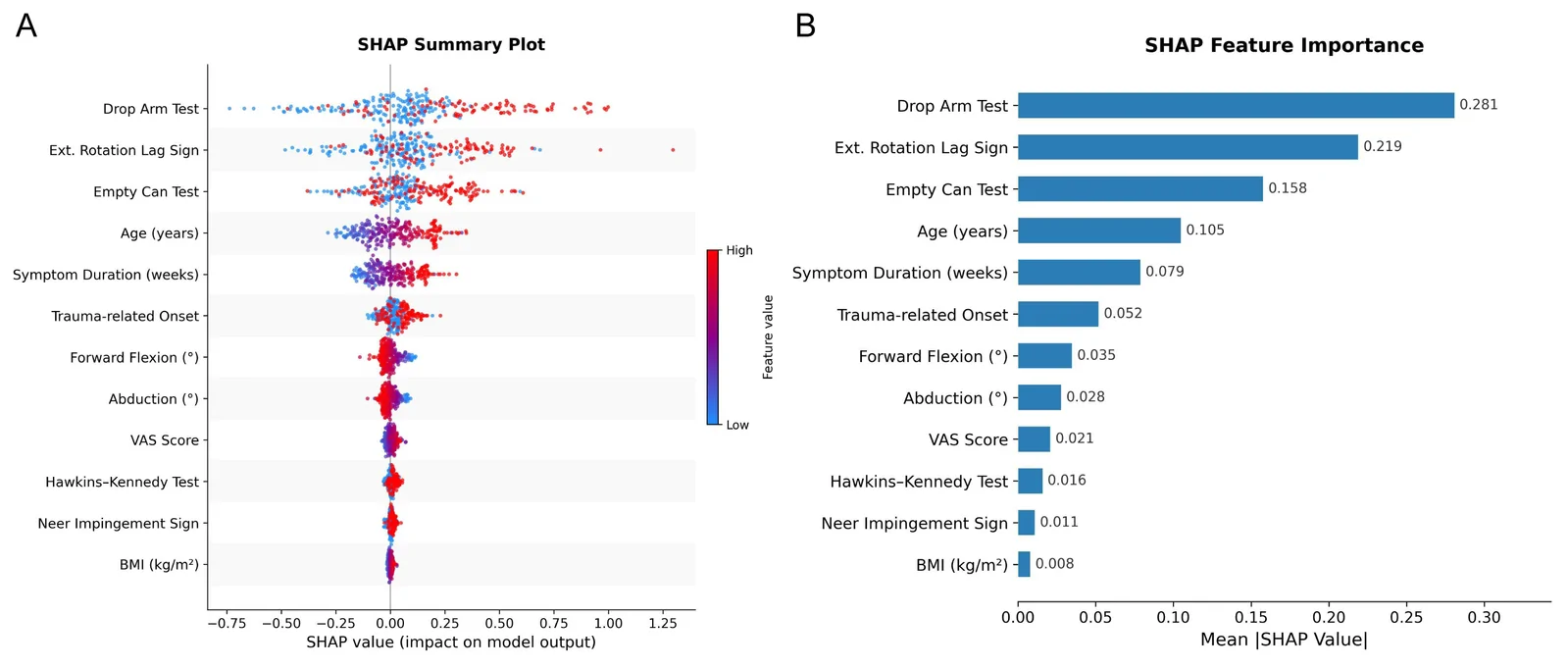
SHAP-based interpretation of the clinical encoder on the testing set. **(A)** Beeswarm plot showing the magnitude and direction of each feature’s contribution, with points colored by feature value. **(B)** Mean absolute SHAP values ranked by overall importance.

**Figure 4:**
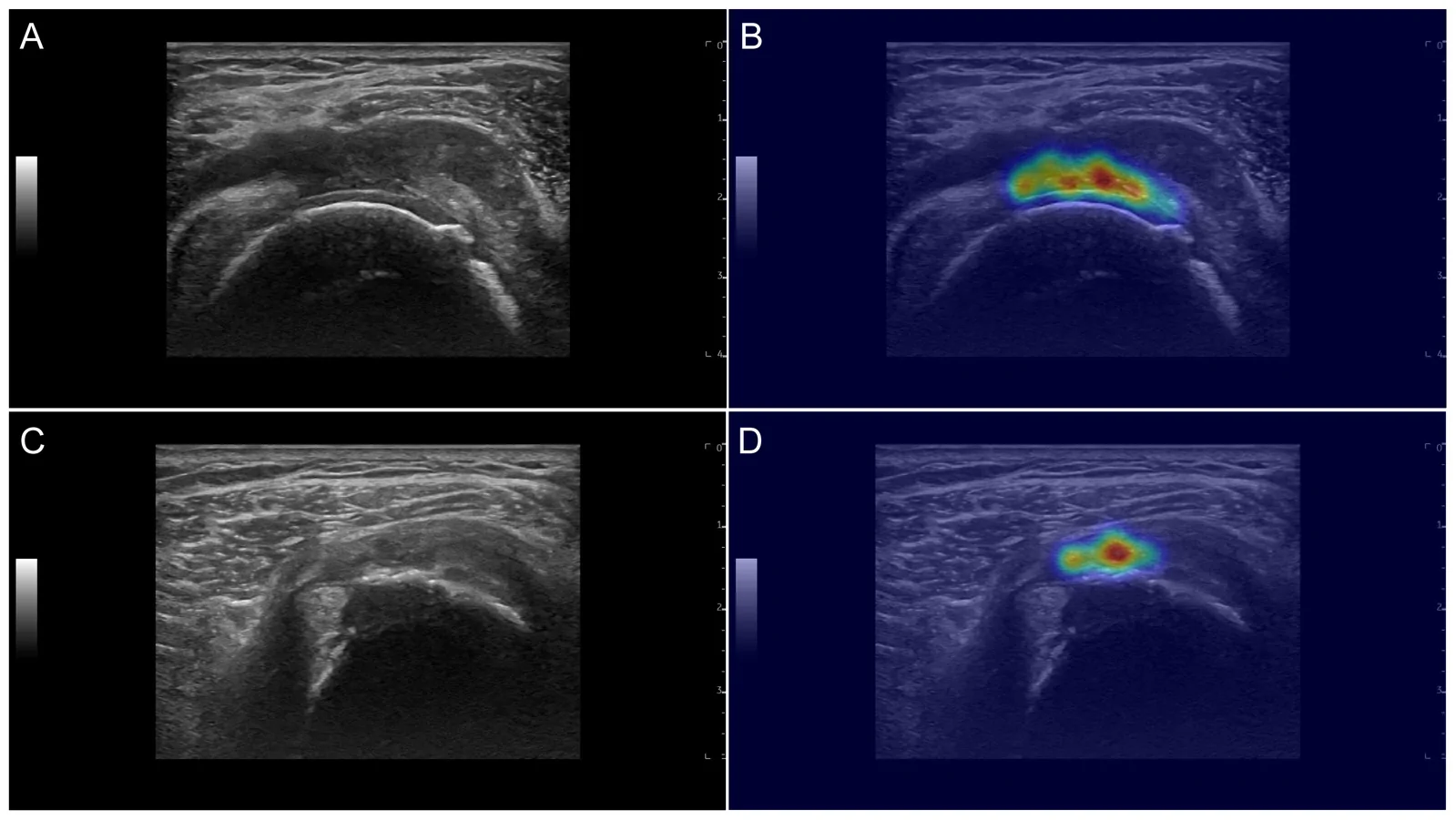
Grad-CAM heatmaps superimposed on representative US images from an FTRCT case **(A, B)** and a PTRCT case **(C, D)**. Activation in the FTRCT case spans broadly across the tendon–bone interface, whereas that in the PTRCT case is focally concentrated at the articular surface defect. Warmer colors denote greater contribution to the model prediction.

SHAP analysis identified the drop arm test, external rotation lag sign, and empty can test as the three most influential clinical features, collectively dominating the clinical encoder output (**Figure 3**). For all three tests, positive findings corresponded to higher SHAP values, indicating increased predicted probability of RCT, consistent with their established clinical significance for detecting rotator cuff pathology. Age, symptom duration, and trauma-related onset contributed at an intermediate level, while range-of-motion parameters, VAS score, and general impingement signs exerted comparatively modest influence. Grad-CAM visualization suggested that image encoder attention was spatially concentrated within the supraspinatus tendon region across both RCT subtypes, consistent with anatomically plausible feature extraction (**Figure 4**). In FTRCT, activation spanned broadly across the tendon–bone interface, consistent with diffuse structural disruption, whereas in PTRCT it appeared more focal, in a pattern consistent with a smaller, localized lesion. These findings suggest that model attention was directed toward sonographically relevant regions rather than background noise or image artifacts.

## Discussion

Accurate sonographic identification of RCTs remains an unresolved clinical challenge due to the intrinsic limitations of single-modality US and the irreducible influence of operator experience. In the present study, we developed and validated a multimodal DL framework that jointly leveraged US imaging features and clinical variables for RCT detection, using MRI as the reference standard. The integrated MM model demonstrated consistently superior diagnostic performance across all evaluated metrics compared with any unimodal configuration, achieving a clinically meaningful balance between sensitivity and specificity that neither imaging nor clinical data alone could attain. To our knowledge, this represents one of the first studies to systematically combine US with clinical examination findings within a unified DL architecture for RCT identification. Unlike prior investigations that treated imaging and clinical modalities as independent diagnostic tools, our feature-level fusion strategy enabled complementary signal integration at the representation level, yielding a model whose predictions were both quantitatively superior and anatomically interpretable. These findings carry direct clinical relevance, as the proposed framework offers a viable pathway toward MRI-independent RCT diagnosis in resource-constrained settings where advanced imaging remains inaccessible or impractical.

### Methodological innovations

The present study employed a feature-level fusion strategy, integrating imaging and clinical representations at the learned embedding level prior to joint classification rather than combining unimodal predictions at the decision stage. This intermediate fusion paradigm enables cross-modal interactions to be captured during end-to-end training, allowing the two branches to mutually constrain each other’s representations in a manner that decision-level ensembling cannot replicate (Li et al., 2024). This design choice contrasts with ensemble approaches previously applied to related musculoskeletal imaging tasks, such as the late-stage combination of independently trained radiograph-based and clinical models for rotator cuff pathology classification (Alike et al., 2023); such ensembling cannot capture cross-modal interactions during training and may therefore underuse complementary signal when neither modality alone is strongly discriminative, as is the case for structured clinical variables in RCT diagnosis. To our knowledge, no prior study has applied a feature-level multimodal deep learning framework integrating US imaging with clinical variables specifically for RCT diagnosis. The motivation for incorporating clinical variables alongside US imaging was rooted in the recognized diagnostic ceiling of single-modality US, particularly for PTRCTs. Encoding physical examination findings and demographic parameters alongside image-derived features exploited complementary signal dimensions inaccessible to imaging alone, consistent with prior evidence that integrating clinical data with deep visual features yields superior performance over either source individually in tendon pathology detection (Chiu et al., 2022; Alike et al., 2023). To address the translational concern that high-performing DL models may remain clinically unacceptable due to their opacity, the present framework incorporated interpretability tools spanning both modalities. Grad-CAM provided spatially resolved heatmaps indicating that model attention was concentrated within sonographically relevant structures, though such maps alone cannot confirm precise pathological localization or exclude reliance on non-target structures without formal expert assessment or quantitative localization analysis. SHAP quantified the direction and magnitude of each clinical variable’s contribution to model output, offering feature-level transparency consistent with established recommendations for XAI implementation in musculoskeletal AI (Glinkowski et al., 2026). Together, these mechanisms reduce dependence on black-box inference and offer clinicians mechanistic insight that may support evaluation of model predictions in practice.

### Interpretation of model performance

The MM model demonstrated consistently superior diagnostic performance across all evaluated metrics, with statistically significant improvements over every unimodal configuration. Prior DL approaches applied to US-based rotator cuff detection have reported variable performance across architectures and study populations (Zhan et al., 2024); a recent attention-augmented single-view model achieved improved detection accuracy over its baseline counterpart yet remained constrained by the absence of clinical contextual data (Wu et al., 2025), its high reported accuracy was obtained on a simplified binary object-detection task performed on a class-balanced image-level cohort, a setting known to yield inflated apparent accuracy relative to prevalence-based, patient-level, multimodal clinical prediction tasks such as the one addressed here. The present findings suggest that these limitations are meaningfully overcome through multimodal integration. The negligible gain from single- to dual-view US encoding in the present cohort suggests that the primary view already captured the sonographic features most relevant to supraspinatus tear classification, in contrast to prior work in musculoskeletal ultrasound reporting improved performance with combined-view over single-view models (Chiu et al., 2022), indicating that the marginal value of multi-view encoding may be task- and pathology-dependent; by comparison, the more substantial improvement conferred by adding clinical variables confirms that physical examination findings encode complementary predictive signal inaccessible to imaging alone. This pattern is consistent with prior work showing that tendon-specific provocation tests dominate SHAP-based feature importance in clinical-only machine learning models for RCT prediction (Li et al., 2023). However, the present clinical encoder achieved more modest discrimination than the accuracy and AUC ranges reported in that study, likely because the present broader ultrasound-referred cohort spans a wider spectrum of milder, clinically overlapping presentations than the more selected surgical cohort in that study, making clinical-only discrimination inherently more difficult. The clinical encoder in the present framework reproduced this hierarchy, prioritizing the drop arm test, external rotation lag sign, and empty can test, an ordering mechanistically consistent with the established high specificity of rotator cuff lag signs for posterosuperior pathology and the convergent validity of the empty can test for supraspinatus involvement (Jain et al., 2017; Yazigi Junior et al., 2021), thereby supporting the biological plausibility of the model’s decision logic. The imaging-only configurations achieved comparatively lower specificity than MM, attributable to the absence of contextual information capable of disambiguating sonographically overlapping pathologies. Incorporation of clinical variables improved both sensitivity and specificity, yielding a clinically viable balance between the two with direct relevance to triage settings where both missed tears and unnecessary surgical referrals carry meaningful consequence. Importantly, subgroup analysis stratified by tear severity indicated that this overall sensitivity was not driven primarily by the more readily detectable full-thickness tears. All FTRCTs were correctly identified, and although the model’s few false negatives were confined to PTRCTs, sensitivity for this diagnostically more challenging subtype remained well above the median sonographic sensitivity of approximately 0.65 reported for partial-thickness tears on conventional US (Farooqi et al., 2021; Radhakrishnan et al., 2024). Because PTRCTs are both more prevalent and more frequently missed on single-modality US, this preserved sensitivity for the harder-to-detect subtype suggests that integrating clinical findings with US imaging helps recover partial-thickness cases that imaging alone would overlook, reinforcing the clinical value of the multimodal approach. This clinical utility was corroborated by the DCA; however, because calibration was imperfect, the specific threshold probabilities evaluated may not correspond precisely to true risk, and the relative ranking of net benefit across configurations should be considered more robust than the absolute values.

### Clinical implications

The present framework offers a practical pathway toward MRI-independent rotator cuff diagnosis, particularly in primary care settings across China where MRI availability remains unevenly distributed. Operating entirely within existing point-of-care resources, the model requires no additional investigations or infrastructure beyond routine US examination and standard clinical assessment. In such contexts, a validated decision-support tool of this kind may reduce unnecessary referrals while expediting surgical triage for patients with confirmed structural pathology. The interpretability mechanisms embedded within the framework further facilitate clinical translation, as Grad-CAM heatmaps offer sonographers a qualitative visualization of the image regions contributing to each prediction, while population-level SHAP feature rankings remain directly legible to clinicians, offering a potential mechanism for human oversight and responsible AI deployment in diagnostic practice (34). The modular encoder architecture additionally permits targeted retraining on institution-specific datasets, supporting adaptation across heterogeneous clinical environments without full model reconstruction.

### Limitations and future directions

Several limitations of the present study warrant acknowledgment. First, the single-center retrospective design restricts generalizability, and whether the model retains its discriminative performance across different operators, equipment configurations, and patient populations remains to be established through external validation. Second, the model’s diagnostic scope was narrow in two respects. It addressed supraspinatus pathology exclusively, leaving the framework’s utility for infraspinatus, subscapularis, and teres minor tears uncharacterized. It was also developed and evaluated against a restricted set of controls limited to supraspinatus tendinopathy and subacromial bursitis, such that its generalizability to broader clinical populations presenting with shoulder pain from other rotator cuff or non-rotator cuff pathologies remains unestablished. Third, physical examination findings were recorded based on individual clinician judgment without formal quantification of interrater reliability, introducing a potential source of measurement variability that may have influenced clinical encoder performance. Fourth, although missing values were rare and single imputation was selected to retain the full analytical cohort without introducing additional model-based assumptions, median and mode imputation does not account for uncertainty in the imputed values. Therefore, a modest influence of the imputation strategy on model estimates cannot be fully excluded, although this effect was unlikely to materially affect the study findings given the low proportion of missing data. Fifth, the image encoder processed static sonographic frames; incorporating dynamic US sequences may capture diagnostic information inaccessible from still images alone. In addition, although 224 × 224-pixel inputs facilitated standardized and computationally feasible training, this resolution may have reduced fine-grained sonographic details relevant to small partial-thickness tears. Similarly, interpretability was assessed separately for each modality rather than through explicit analysis of cross-modal interactions. Sixth, although a stratified subgroup analysis showed that sensitivity was lower for PTRCTs than for FTRCTs while remaining high, this comparison was based on a modest number of PTRCTs from a single center, and larger multicenter cohorts are needed to confirm consistent performance across tear severities. Future work should prioritize prospective multicenter validation alongside extension of the framework to full rotator cuff assessment. Formal reliability quantification of clinical examination variables, comparison of alternative input resolutions, exploration of architectures capable of processing dynamic US sequences, and interpretation of cross-modal interactions represent further directions through which the robustness and diagnostic scope of the model may be advanced.

## Conclusion

In conclusion, this study developed and validated a multimodal DL framework integrating US imaging with clinical data for RCT detection, using MRI as the reference standard. The multimodal model demonstrated superior diagnostic performance over all unimodal configurations, achieving a clinically meaningful balance between sensitivity and specificity that neither imaging nor clinical data alone could attain. These findings suggest that multimodal DL holds potential as a viable and accessible strategy for MRI-independent rotator cuff diagnosis, with particular relevance to resource-constrained settings where advanced imaging remains unavailable, pending external validation across broader patient populations and shoulder pathologies.

## Data Availability

The original contributions presented in the study are included in the article/**Supplementary Material**. Further inquiries can be directed to the corresponding author.
