# Supplementary material for: Multimodal deep learning integrating ultrasonographic and clinical data for enhanced rotator cuff tear identification
Source: Front Physiol. 2026 Sep 11;17:1884047. doi: 10.3389/fphys.2026.1884047 (PMC13612273; doi:10.3389/fphys.2026.1884047)
Supplement: Supplementary file 1 [file Table1.docx]

**Supplementary**

**Table S1**. Baseline characteristics of the training and testing sets

| Variable | Training set  (n = 663) | Testing set  (n = 284) | *P* value |
| --- | --- | --- | --- |
| RCT-positive, n (%) | 259 (39.1) | 111 (39.1) | 1.000 |
| Age, years | 55.2 ± 10.5 | 55.6 ± 10.2 | 0.577 |
| Male sex, n (%) | 311 (46.9) | 133 (46.8) | 0.984 |
| BMI, kg/m² | 24.8 ± 3.3 | 24.8 ± 3.3 | 0.941 |
| Right side affected, n (%) | 432 (65.2) | 185 (65.1) | 0.989 |
| Symptom duration, weeks | 16 (8–34) | 16 (8–36) | 0.742^a^ |
| Trauma-related onset, n (%) | 162 (24.4) | 68 (23.9) | 0.853 |
| VAS pain score | 5.5 ± 1.9 | 5.5 ± 1.9 | 0.962 |
| Forward flexion, ° | 148.2 ± 21.9 | 147.4 ± 21.5 | 0.619 |
| Abduction, ° | 140.2 ± 24.3 | 139.4 ± 24.0 | 0.634 |
| Internal rotation, ° | 51.3 ± 12.2 | 51.0 ± 12.2 | 0.720 |
| External rotation, ° | 54.9 ± 13.7 | 54.5 ± 13.6 | 0.680 |
| Neer impingement sign positive, n (%) | 439 (66.2) | 188 (66.2) | 0.999 |
| Hawkins–Kennedy test positive, n (%) | 466 (70.3) | 199 (70.1) | 0.948 |
| Empty can test positive, n (%) | 324 (48.9) | 139 (48.9) | 0.994 |
| Drop arm test positive, n (%) | 64 (9.7) | 28 (9.9) | 0.924 |
| External rotation lag sign positive, n (%) | 52 (7.8) | 23 (8.1) | 0.872 |

^a^compared using the Mann–Whitney U test
